# Impact of River Morphology on River–Groundwater Exchange in Braided River Systems

**DOI:** 10.1111/gwat.70092

**Published:** 2026-07-15

**Authors:** Moritz Kraft, Antoine Di Ciacca, Thomas Wöhling

**Affiliations:** ^1^ Chair of Hydrology Dresden University of Technology (TUD) 01069 Dresden Germany; ^2^ Formerly Lincoln Agritech Ltd. Ruakura Research Centre Hamilton 3240 New Zealand; ^3^ Currently at Centre for Hydrogeology and Geothermics Université de Neuchâtel 2000 Neuchâtel Switzerland

## Abstract

Braided river systems are an important source for groundwater recharge, but their complex morphology makes river–groundwater exchange fluxes difficult to estimate. Their river channel morphology changes frequently after floods, which has effects on recharge rates that have rarely been studied in the past. This work aims to isolate the effects of changes in braided river morphology on groundwater recharge for two sections of the Wairau River and Waikirikiri River in New Zealand. For each study site, two different river morphology variants of a fully coupled surface water–groundwater model utilizing high‐resolution DEMs of river bathymetry before and after a major flood event were set up while keeping parameterization and boundary conditions the same. The models demonstrate that flood‐induced morphology changes in braided river systems alter groundwater recharge. We identify features, both simulated and observed, that explain the direction of change. Features that increase groundwater recharge are a larger braidplain aquifer extent and volume, larger wetted area and, specifically, an increase of areas with high exchange rates in locations of larger gradients between braidplain aquifer and regional aquifer. These factors influence groundwater recharge independent of connection (Wairau River) or disconnection (Waikirikiri River) of the system to the regional aquifer, albeit with different magnitudes. An extension of our research to other braided rivers is needed to more broadly generalize our findings.

## Introduction

The complex interplay between rivers and groundwater is an ongoing field of research for many decades in both hydrological and hydrogeological sciences. This holds especially true in areas where groundwater recharge from river losses or river flow gains from groundwater discharge into the river are major system drivers. Hydrogeologically, a major topic of research has been the focus on river–groundwater exchange as a potential recharge factor for aquifer systems (Fenemor 1989; Stanley and Jones 2000; Jones et al. 2008; Larned et al. 2008; Baalousha 2012; White et al. 2012; Close et al. 2014; Yang et al. 2017; Wöhling et al. 2020; Di Ciacca et al. 2024; Wilson et al. 2024). Research on rivers as important drivers of aquifer recharge has many different directions: estimations of recharge (Close et al. 2014; González‐Pinzón et al. 2015), classification of system dynamics at different scales (Dahl et al. 2007), measurement methods (Kalbus et al. 2006; Rosenberry and LaBaugh 2008; Lamontagne et al. 2014; González‐Pinzón et al. 2015) or the analysis of the mechanics of river–groundwater interaction (Brunner et al. 2009a, 2009b, 2011; Wöhling et al. 2020; Wilson et al. 2024).

Regarding the dependencies of river–groundwater interaction on a regional level, classification of the connection, or disconnection, between river and regional aquifer has been identified as a major factor (Brunner et al. 2009a). Generally, states are differentiated between connected, where groundwater levels are at or above the river bed; disconnected, where an unsaturated zone between riverbed and aquifer exists and changes in groundwater levels do not affect exchange fluxes; and transitioning states between the two endpoints. Viewing exchange as a regional recharge driver, this is especially important, as recharge is independent of aquifer water levels in disconnected systems (Brunner et al. 2011), while in connected systems, river water and groundwater levels both affect the exchange rate, and discharge from the groundwater into the surface systems is also possible (Brunner et al. 2009a). Therefore, disconnected rivers are always “losing” rivers, while connected rivers can be losing or gaining, depending on the gradient between groundwater level and river level.

Due to the difficulty of measuring these phenomena in the field (Kalbus et al. 2006; González‐Pinzón et al. 2015), modeling river–groundwater interaction is a major factor in describing, analyzing, and potentially estimating the interplay between surface and subsurface. Besides many modeling studies describing specific systems (Fenemor 1989; Brown et al. 1999; Baalousha 2012; White et al. 2012; Maxwell et al. 2015; Yang et al. 2017; Wöhling et al. 2020), research is undertaken in the integration of interaction in existing models, mainly in fully coupled models (LaBolle et al. 2003; Panday and Huyakorn 2004; Kollet and Maxwell 2006; Furman 2008; Jones et al. 2008; Sudicky et al. 2008; Spanoudaki et al. 2009; Brunner et al. 2010; Barthel and Banzhaf 2016; Di Ciacca et al. 2024). In model frameworks focusing on the description of subsurface systems, such as MODFLOW (?) or PARFLOW (Maxwell et al. 2009), rivers are treated as boundary conditions of the aquifer systems (Kollet and Maxwell 2006; Brunner et al. 2010; Barthel and Banzhaf 2016; Wöhling et al. 2020). Thus, their representation in the model is simplified and might result in erroneous exchange simulations due to scale differences, missing processes, and different parameterization, among other factors. With increasing computational power, fully coupled surface–subsurface models such as HydroGeoSphere (Brunner and Simmons 2012) have been applied to more numerous and varied cases (Spanoudaki et al. 2009; Barthel and Banzhaf 2016; Di Ciacca et al. 2024). While these models usually lead to a more realistic representation of the river system, and thus the interplay with the subsurface (Barthel and Banzhaf 2016), new problems such as difficulties of parameterization or soaring computational expenses arise.

**Figure 1:**
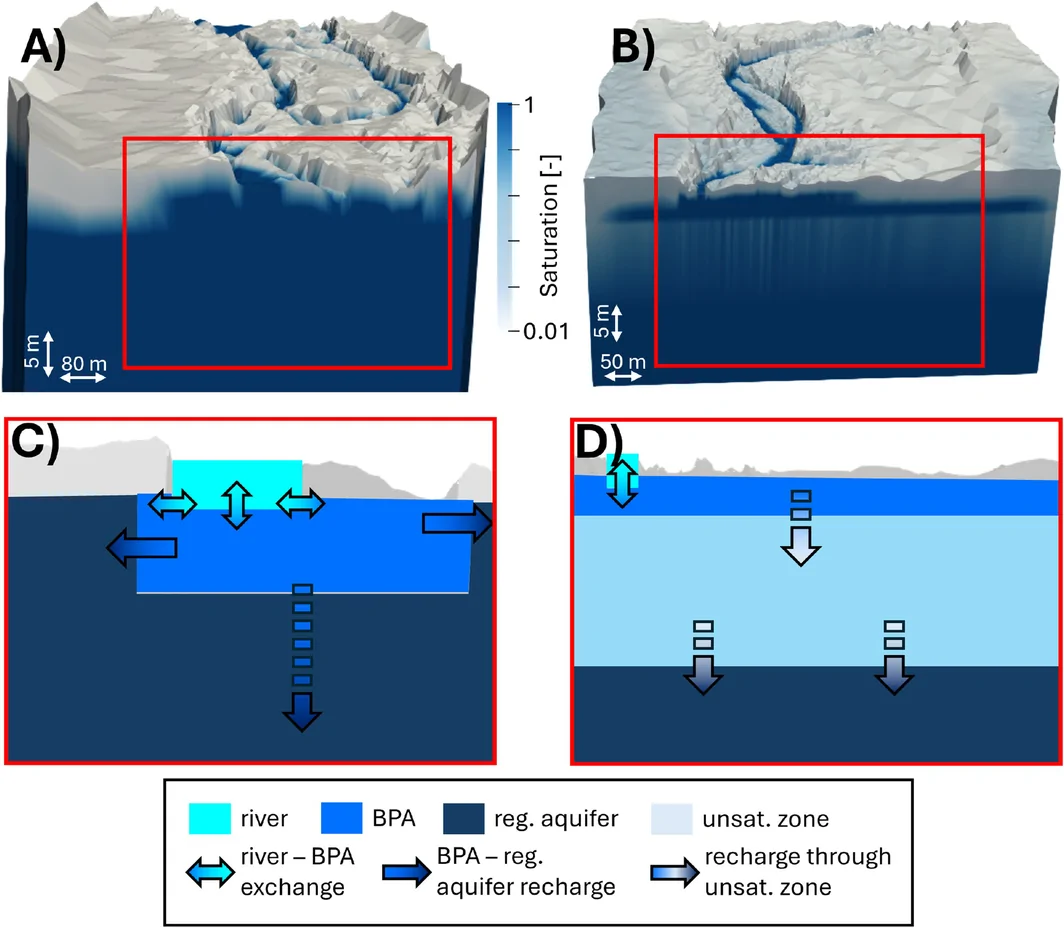
Conceptual overview of braidplain aquifer (BPA) topology and interaction. Top row shows exemplary cross‐sections of saturation for the two models in this study: (A) Wairau River, BPA connected to regional aquifer, and (B) Waikirikiri River, BPA disconnected from regional aquifer. Bottom row shows the corresponding conceptual cross‐sections of BPA systems connected to (C, Wairau River) and disconnected from (D, Waikirikiri River) the regional aquifer, with arrows indicating the main pathways of river to groundwater exchange/recharge (light to medium blue), BPA to regional aquifer exchange (medium to dark blue) and the intermediate pathway through the unsaturated zone (medium to light blue to dark blue, only disconnected).

Furthermore, different types of river systems bring their own unique factors and challenges. Braided rivers are dynamic rivers carrying large sediment loads, leading to the formation of gravel beds that are often reworked by their branching and meandering rivers (Charlton 2007). These properties of braided rivers influence the aquifer systems in unique ways, creating alluvial, heterogeneous aquifers with many preferential flow paths formed by old river channels, which underlie the active river beds (Huggenberger and Regli 2006). This leads to the formation of distinct, shallow alluvial aquifers with a strong interaction with the river (Stanford and Ward 1993; Poole and Berman 2001; Boano et al. 2008). These aquifers, termed braidplain aquifers in more recent research (Di Ciacca et al. 2024; Wilson et al. 2024), interact strongly with the river system, essentially acting like a subsurface extension of the river where parafluvial and hyporheic flow takes place. The braidplain aquifer (or BPA in short) acts as a buffer between the spatially and temporally highly dynamic river–BPA interactions and typically less dynamic interactions between the BPA and the regional groundwater. Furthermore, the BPA can even be found to be geologically separated from the regional aquifer by a distinct geological change (Wilson et al. 2024). Instead of a direct connection between river and regional aquifer, river and BPA are interacting directly (BPA recharge), while exchanges between BPA and the regional aquifer (regional aquifer recharge) depend on their connection: following the usual conceptualization of river–groundwater exchange defined above, BPA and regional aquifer can be connected, disconnected or in an intermediate state (Figure 1). The concepts of gaining or losing rivers can then be further recontextualized in regards to BPA–regional aquifer connection: a system with a regional aquifer disconnected from the BPA is always losing (to the regional aquifer), while for a connected system the local (spatial and temporal) head gradients between BPA and regional aquifer define whether the system is gaining or losing. In these systems, researchers are studying the formation and estimation of channel and braidplain heterogeneity (Guin et al. 2010; Ramanathan et al. 2010; Javernick et al. 2016; Zovi et al. 2017) and the unique interactions between river and groundwater (Larned et al. 2008; White et al. 2012; Banks et al. 2022; Di Ciacca et al. 2024, 2025; Sai Louie et al. 2024; Wilson et al. 2024). Furthermore, the morphological changes in the braidplain channels and riverbed architecture due to frequent flooding events have been studied, with a focus on describing morphology changes in models (Pirot et al. 2014; Williams et al. 2014, 2016). While Sai Louie et al. (2025) studied the changes on water fluxes at a braidplain cross‐section using A‐DTS sensing, the isolated impact of braided river morphology on magnitudes and relative changes of river–groundwater exchange fluxes, however, has not yet been investigated.

This study addresses this gap by setting up twin model variants for sections of the Wairau River and the Waikirikiri Selwyn River on the South Island of New Zealand. Highly detailed, fully coupled surface–subsurface models were set up in HydroGeoSphere using Lidar‐derived DEMs taken before and after a major flood event in the two rivers. Apart from the river bathymetry, the two model variants of each site are otherwise identical, which allows investigating for the first time the change of both the net groundwater exchange fluxes and their spatial patterns due to naturally occurring shifts in riverbed morphology. This study addresses the following research question: Do river–groundwater interaction rates in braided rivers change as a result of changes in riverbed morphology? If so, what are the most influential hydro‐morphological features for that change?

## Materials and Methods

This section starts with an abbreviated description of the two study areas and the differences of their braided river systems. This includes an overview of the available data for the two study areas, incorporating both hydrogeological measurement time series as well as the morphological data. Then, both models are described, from boundary condition setup over calibration to the model variants used to compare river–groundwater interaction under morphological changes. Throughout this section, important differences between the two study areas and their models are highlighted. Note that both the Wairau River and Waikirikiri River study sites are already described in Wilson et al. (2024) in more detail for interested readers.

### Study Areas and Data

#### Wairau River

**Figure 2:**
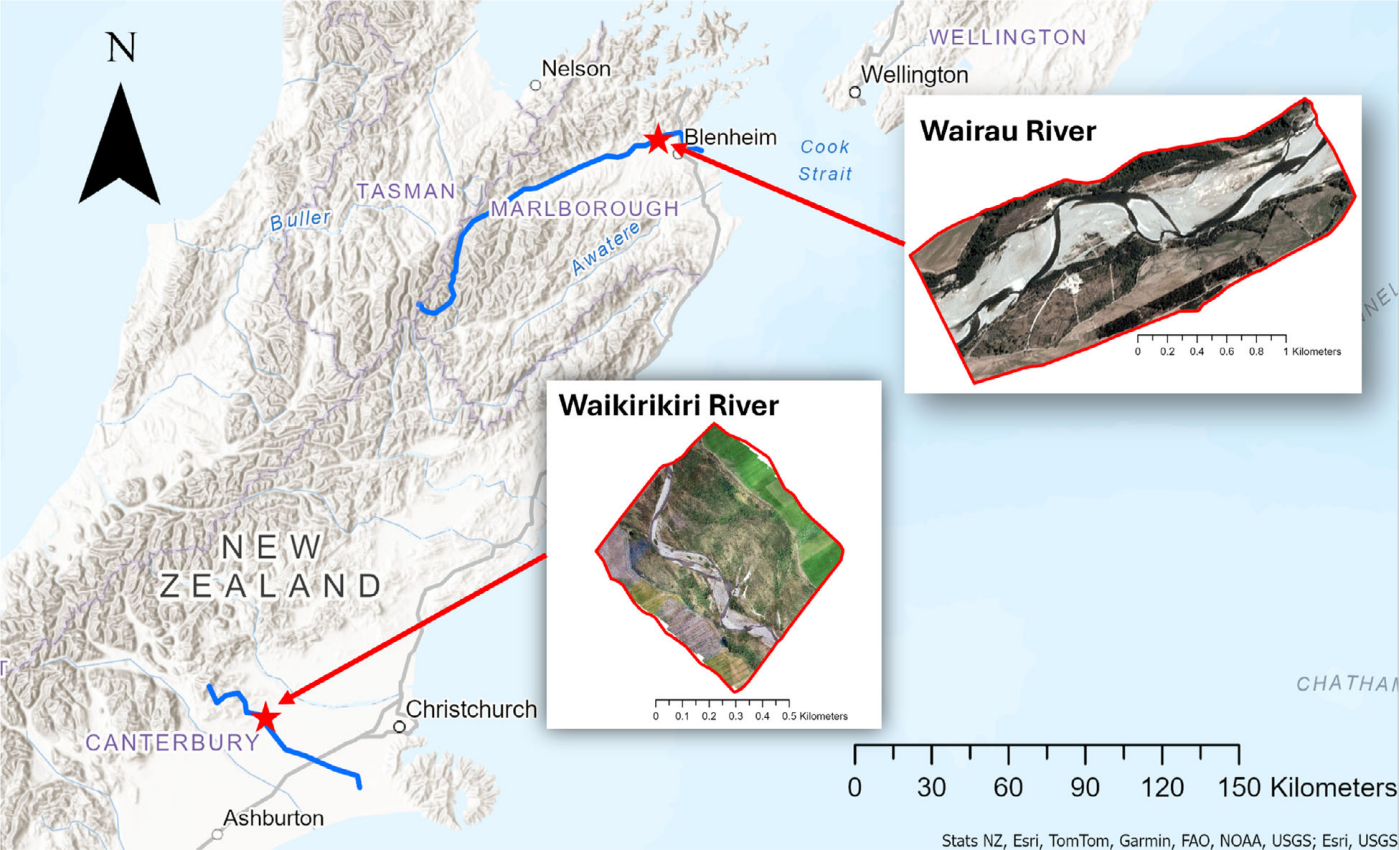
Location of Wairau River (date of aerial photo: February 24, 2020) and Waikirikiri River (date of aerial photo: January 8, 2020) study sites in New Zealand.

The Wairau River has a length of about 170 km, originating in the Spenser Range in the north of the Southern Alps of New Zealand and ends in the Pacific ocean near the city of Blenheim (Figure 2). The river crosses the Wairau Plains northwest of Blenheim, which is underlain by the Wairau Aquifer, a major source of freshwater for the region mainly recharged by the Wairau River. Along the Wairau Plains, the river goes from perched unconnected several meters above the aquifer in the west through a transitioning state to a fully connected state in the east, where a wedge of fine marine sediments increasingly confine the Wairau Aquifer, forcing several springs to emerge. In this area, based on over 60 years of recorded flow at SH1, the Wairau River has a mean flow of about 100 m^3^/s and a peak flow of more than 5000 m^3^/s, with several high flow events annually. Due to the importance of the river–groundwater interaction for regional aquifer recharge, the system has been the focus of many past and recent studies (Brown 1981; Davidson and Wilson 2011; Raiber et al. 2012; Wöhling et al. 2018, 2020, 2025; Gosses and Wöhling 2021; Ejaz et al. 2023; Wilson et al. 2024) with detailed flow‐loss gauging campaigns, groundwater measurement wells and a highly detailed regional‐scale groundwater model developed using MODFLOW that has been in development since the mid‐2010s. (Wöhling et al. 2018, 2025).

**Figure 3:**
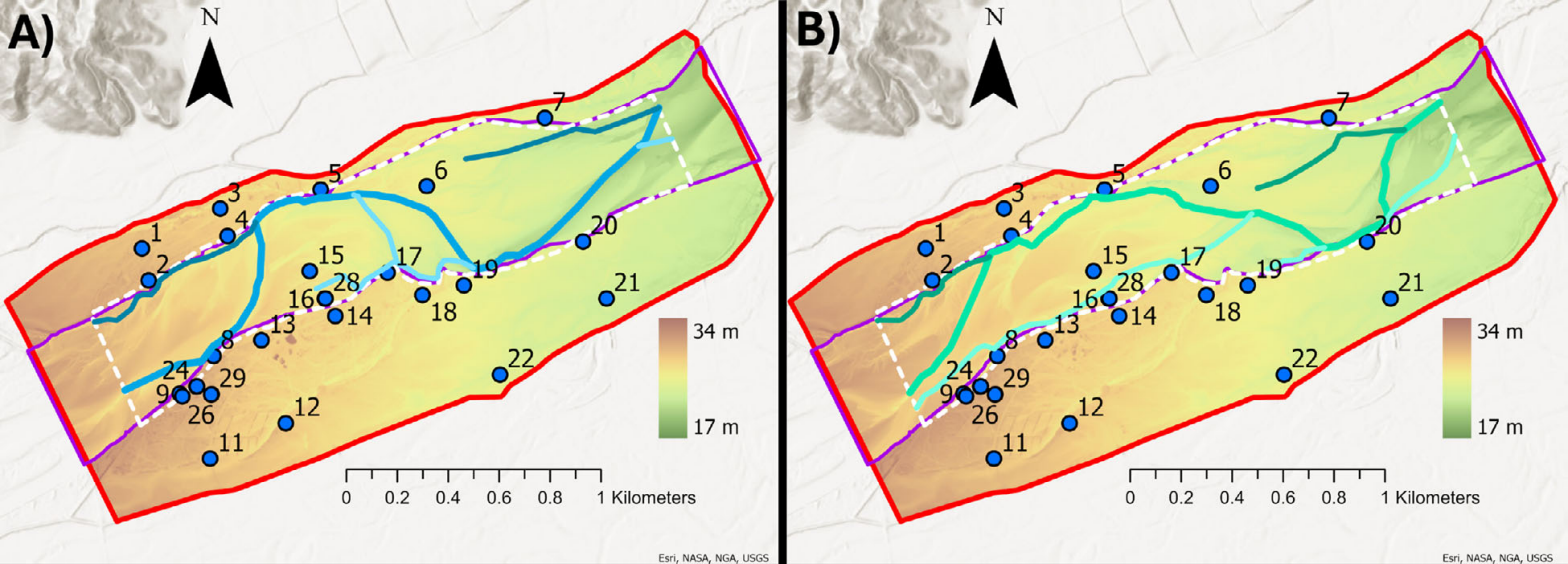
Wairau River model study area with preflood (A, blue river channels) and postflood (B, green river channels) DEMs, along with model extent (red), braidplain extent (purple), analysis subdomain (white dashed), piezometer locations (blue circles) and main (bold), northern (dark), and southern (light) secondary river channels.

The study reach of about 2.2 km length of the Wairau River utilized in this study lies in the middle section of the Wairau Plains, extending roughly from Jeffries Road in the west to Giffords Road in the east (Figure 2). Here, the active braidplain is confined between engineered banks and the river has between one and two major channels during most times. Hydrogeologically, the braidplain aquifer consisting of poorly sorted, unconsolidated gravels overlays the more consolidated, very poorly sorted gravels with increasing silt and clay content of the regional Wairau Aquifer, clearly separated in drill cores (Figure 1, Wilson et al. (2024)). A further, large number of measurements of different types, such as differential flow gaugings, core sediment analyses and river and groundwater levels, have been made in this study site, as detailed in Wilson et al. (2024). For this study, of particular interest are the two high spatial resolution (1 m or less) digital elevation models (DEMs) that represent river bathymetry and braidplain geometry before (preflood, taken on February 18, 2020) and after (postflood, taken on March 31, 2022) a major flood event that occurred on 18.07.2021 with over 5000 m^3^/s peak flow (see Figure 3). The Wairau River is gauged approx. 8 km downstream at State Highway 1, with data available at a 15 min interval. Furthermore, 27 piezometers provide groundwater head measurement data, though not all had data available both pre‐ and postflood as some were installed only after the flood event.

#### Waikirikiri River

The Waikirikiri River flows for 93 km from the foothills of the New Zealand Southern Alps across the alluvial Canterbury Plains to Te Waihora/Lake Ellesmere and the Pacific Ocean (Figure 2). The river course mainly follows a depression between the alluvial fans of the much larger Waimakariri River and Rakaia River. In the foothills, the Waikirikiri River is constrained by hillslopes and has a meandering single‐thread channel. When it reaches the alluvial plains dominated by glacial and periglacial outwash, its flow averages 2 m^3^/s. Past this point, its channel slope decreases abruptly, and it becomes braided or meandering. The Waikirikiri River starts losing water to the underlying aquifers shortly after entering the plains at an average rate of 0.13 m^3^/s/km (Di Ciacca et al. 2025). The transmission losses increase as the water travels through the plains until the river becomes ephemeral (Rupp et al. 2008; Di Ciacca et al. 2024).

The study site is located 10 km downstream after the river enters the plains, at the upstream boundary of the ephemeral reach. The study site is around 750 m long following the river's main flow direction, and the braidplain is around 500 m wide. This reach has been extensively instrumented and studied over the last years by Banks et al. (2022); Di Ciacca et al. (2023); Di Ciacca et al. (2024); Sai Louie et al. (2024, 2025); Wilson et al. (2024). Therefore, only a brief description of the hydrogeological characteristics of this reach is provided here, while referring the reader to the previous studies for more information. The sediments beneath the river are made of an assemblage of glacial and postglacial gravels, which comprise fine to coarse greywacke gravels with occasional cobbles in a fine sand to clay matrix, interbedded with silt layers. They form a complex network of interconnected remnant channels of more permeable gravels surrounded by less permeable sediments. A braidplain aquifer was identified by Banks et al. (2022) and further described by Wilson et al. (2024), and appears to be perched around 10 m above the regional aquifer system (Figure 1). Di Ciacca et al. (2024) demonstrated that this system can be simulated with a three‐layer model, the top layer representing the braidplain aquifer, the bottom representing the regional aquifer, while the middle layer serves as an implicit representation of the intermediate sediment structure described above.

**Figure 4:**
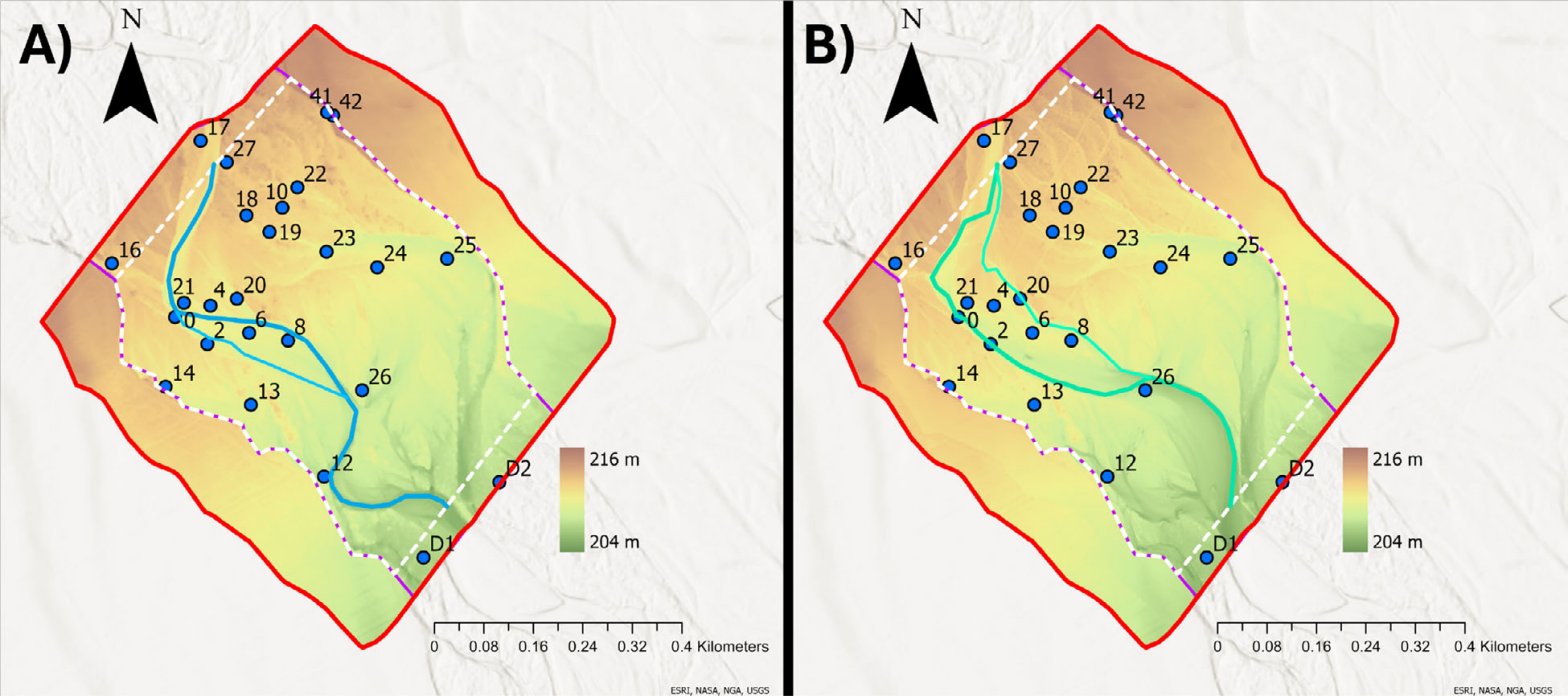
Waikirikiri River model study area with preflood (A, blue river channels) and postflood (B, green river channels) DEMs, along with model extent (red), braidplain extent (purple), analysis subdomain (white dashed), piezometer locations (blue circles) and main (bold) and secondary (light) river channels.

The data used in this study includes two high‐resolution (LiDAR) DEM taken before (preflood, January 8, 2020) and after (postflood, July 2, 2021) a major flood event, which occurred on May 30, 2021 with over 200 m^3^/s of peak flow (Figure 4). During the preflood period, the river was gauged at our study site, however the flood damaged this installation, and the flow at our study site was inferred from the river gauging performed 10 km upstream for the postflood period, as detailed by Di Ciacca et al. (2024). Furthermore, 28 piezometers were used in this study to obtain groundwater head measurement data in the BPA and regional aquifer.

### Modeling Approach

Numerical models were set up for each of the two study sites. In the following we refer to the model for the Wairau River section as WU and the model for the Waikirikiri River section as WI. We further distinguish pre‐ and postflood model variants by numerical labels 1 and 2, respectively. Thus, WU_1_ refers to the Wairau River preflood model and WU_2_ refers to the Wairau River postflood model. Correspondingly, WI_1_ and WI_2_ refer to the Waikirikiri River pre‐ and postflood models, respectively.

Each of the two study sites has its individual model domains, parameterization schemes, boundary conditions etc. as described in the following sections. The two model variants for each river are identical except for their surface elevations: variant 1 utilizes a DEM from before the flood event in the respective rivers, and thus depicts the preflood morphology states. Variant 2 is set up with a DEM taken after the flood event for each of the rivers, describing the postflood morphology in these systems. This setup of model variants allows the isolated investigation of morphology‐related changes of river–groundwater exchange fluxes.

#### Wairau River Model

HydroGeoSphere was used to set up a highly detailed, coupled surface water–groundwater model for the Wairau River study reach. Its final model setup, described in detail below, was arrived at after vigorous testing regarding layering, boundary conditions, convergence and iteration parameters and incorporated hydrogeological information from Wilson et al. (2024).

**Figure 5:**
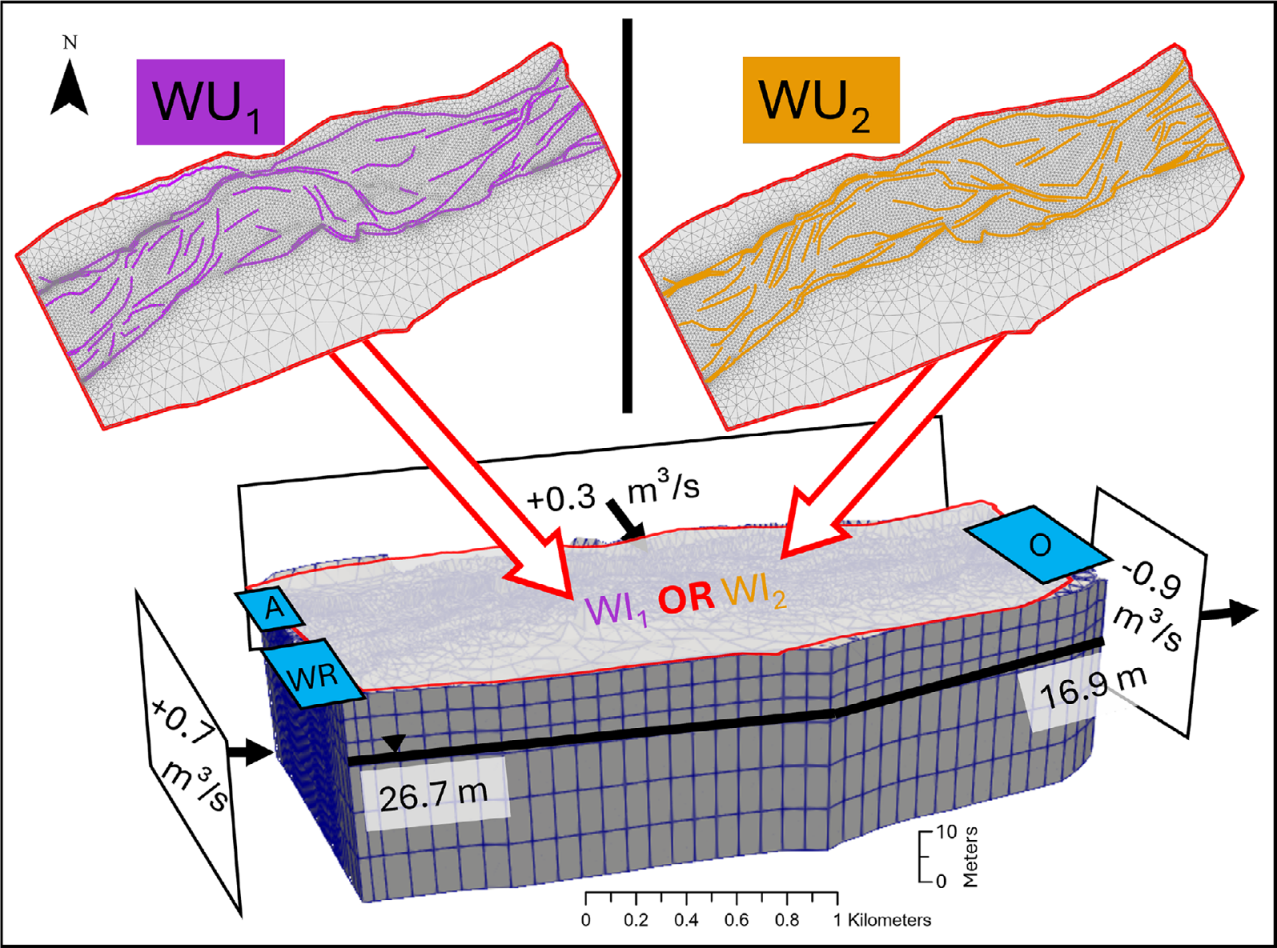
Wairau River model grids and boundary conditions: grid and refinement features are shown for the preflood variant WU_1_ (purple lines and area) and postflood variant WU_2_ (orange lines and area). All other model features are identical: surface model boundaries (blue surfaces) are nodal river inputs (Wairau River [WR] and Are Are Creek [A]) and zero‐depth gradient outflow (O), subsurface model boundaries are western, northern and eastern nodal fluxes (white rectangles) and southern constant head boundary (black line).

The spatial model bounds were determined from the approximate extent of the study area with the Wairau River running from west to east and including enough area outside the northern and southern banks of the braidplain to ensure boundary conditions would not negatively affect model performance (see Figure 5). Vertically, the model was divided into six layers, with three layers dividing the BPA to a depth of about 4.5 m below surface on average, and the other three layers dividing the regional aquifer underneath the BPA to a depth of about 30 m below surface. The contact depth, the surface dividing BPA and regional aquifer was derived from drill cores as described by Wilson et al. (2024).

Hydro(geo‐)logically, the model was parameterized into zones of different properties, with parameters adjusted manually due to the excessive run‐times of the model. On the surface, only two zones are differentiated: (1) the extent of the Wairau River braidplain (purple area in Figure 5) and (2) the land north and south of the riverbanks. The subsurface consists of the regional aquifer material (1), the braidplain aquifer (2) that extends the Wairau River braidplain (purple area in Figure 5) vertically from model top to the third model layer, and (3) an intermediate layer separating BPA and regional aquifer in the fourth model layer. The parameter values chosen in the manual calibration with a multitude of groundwater head measurements (see Model Calibration and Simulation Scenarios below) are detailed in Table 1.

**Table 1:** Hydro(geo‐)logical Model Parameters for the Different Zones of the Wairau River Model

	Subsurface Parameters			Surface Parameters	
Subsurface/Surface Zone	Hydraulic Conductivity *K*_*x*/*y*/*z*_ [ms^−1^]	Porosity Φ [−]	Van Genuchten *S*_*wr*_/*α*/*β* [−/m^−1^/−]	Friction x/y [m^−1/3^s]	Coupling Length [m]
1. Reg. aquifer/reg. land	1 × 10^−3^	0.2	0.011/5/4	0.15	1 × 10^−4^
2. BPA/braidplain	5/5/3 × 10^−3^	0.11	0.011/55/3	0.15	1 × 10^−4^
3. Intermed. layer/—	1 × 10^−5^	0.3	0.011/5/3	—	—

Model boundaries were also split between surface and subsurface (Figure 5). On the surface, the main Wairau River channel on the western model boundary is an inlet of nodal fluxes, utilizing a measured, 15 min interval Wairau River flow time series from the nearest gauging station a few kilometers downstream. Furthermore, a small tributary of the Wairau River, the Are Are Creek, also enters the model as a nodal flux on the north‐western model boundary, again supplied with 15 min interval flow time series from a gauging station. Downstream, the north–south extent of the Wairau River braidplain is modeled as a zero depth gradient type outflow with a bed slope of 0.01.

In the subsurface, constant nodal fluxes were used as boundaries along the western, northern and eastern model edges. The fluxes of +0.7 m^3^/s (west), +0.3 m^3^/s (north), and −0.9 m^3^/s (east) were determined from long‐term mean estimations calculated by inserting the study area into the regional‐scale MODFLOW groundwater model of the Wairau Plains. For the western and eastern subsurface boundaries, the fluxes were divided between regional aquifer and BPA via weighting of the saturated areas. The northern subsurface boundary is only applied to the regional aquifer, as the BPA does not reach the northern model edge. The southern subsurface boundary, which is the major pathway of river recharge to enter the surrounding regional aquifer, was represented as a head boundary. This allows variation in flow across the boundary instead of prescribing a fixed flow value, which allows variable recharge from the river to the wider regional aquifer bordering in the south. The groundwater heads along this boundary were estimated with a model variant featuring steady‐state simulations with mean river flow and a constant southern flux boundary of −1.2 m^3^/s, again taken from the regional‐scale model, and decline from 26.7 m AMSL at the south‐western edge to 16.9 m AMSL at the south‐eastern model edge. As shown above (Figure 3), a total of 27 piezometers provided groundwater head measurements in the study area.

For this study, two model variants (WU_1_ and WU_2_) were utilized, only differing in the DEM that provides the surface elevation of the model (see Figure 5). The horizontal model grid of WU_1_ was created from a preflood DEM taken on 18.02.2020 (by LiDAR), while the grid of WU_2_ was created from a postflood DEM taken on March 31, 2022 (by LiDAR). The grids were generated using Algomesh (?), with a refinement area overlaying the braidplain with maximum element edge lengths of 20 m and line features manually extracted from the DEMs to restrict edge lengths along the river talweg (10 m), secondary channels (20 m), braidplain banks (10 m), and outer model boundaries (80 m) (Figure 5). As a result, both grids consist of over 16,000 triangular elements/over 8000 triangular nodes per model layer.

Note that this setup naturally leads to differences in the exact location of river boundary nodes between the two model variants, because they necessarily represent the changes in morphology between the variants, including at the model boundaries. This river boundary flow, however, is identical for the model variants, and the change in channel node location does not have a noticeable influence on the results presented below. Model simulations to test the sensitivity of river boundary node locations to compute changes in exchange fluxes have shown miniscule differences in model results: resulting changes in piezometer heads and exchange fluxes were found to be less than 0.5% (results not shown), and are thus considered negligible. As a further safety measure to exclude any of these marginal boundary effects, we excluded areas close to upstream and downstream river boundaries from the analysis (c.f. Figures 3 and 4).

#### Waikirikiri River Model

Using the same procedure as for the Wairau River study site, a single 3D integrated surface and subsurface model was set up for the Waikirikiri River site in HydroGeoSphere. Its setup, using the preflood DEM, as well as its parameterization and evaluation, have been described in detail by Di Ciacca et al. (2024). In short, this model represents a section of the river and its braidplain aquifer and extends vertically until the first deeper aquifer. It uses three hydrogeological layers with differing hydraulic properties, which are each divided into two numerical layers. The upstream river flow was specified using the synthetic flow record inferred from the river gauging station located 10 km upstream, as detailed by Di Ciacca et al. (2024).

**Figure 6:**
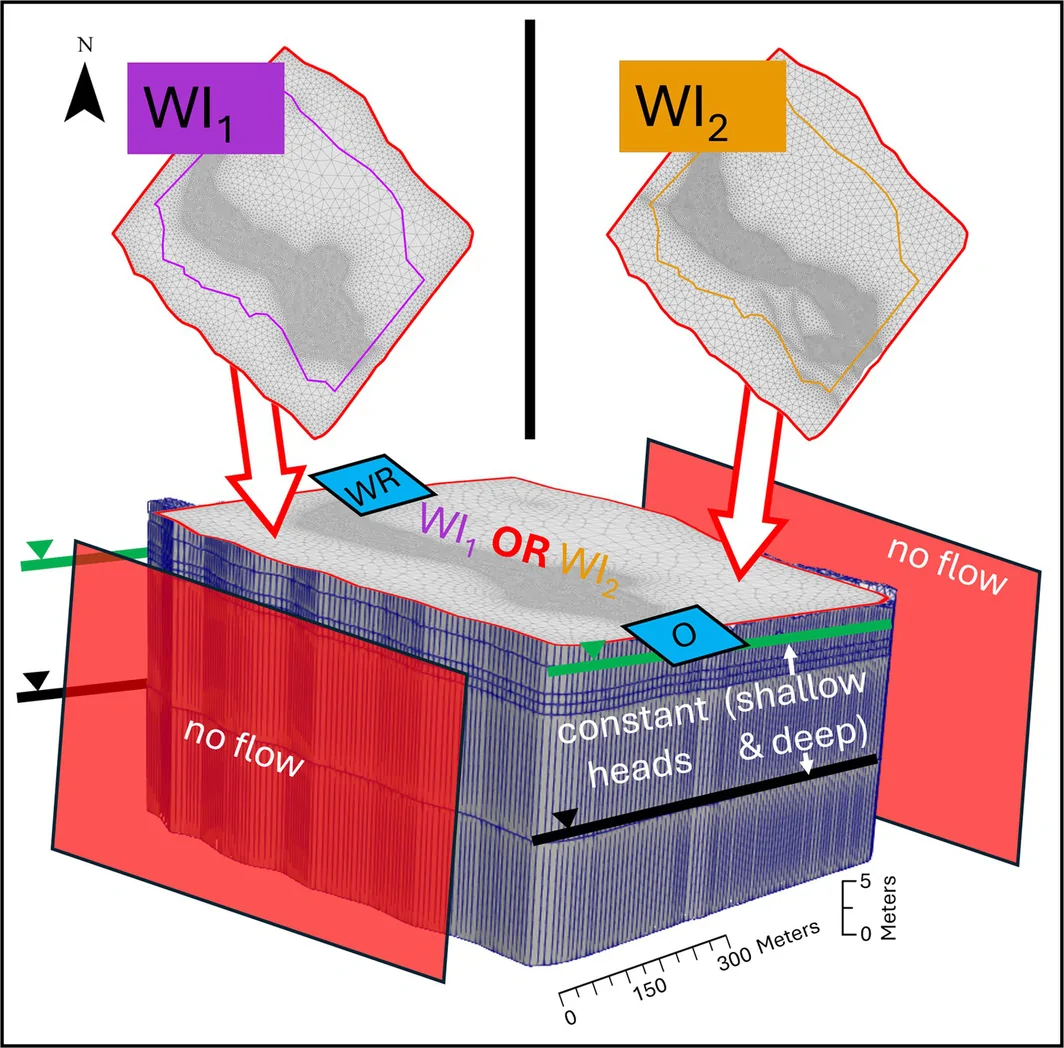
Waikirikiri River model grid and boundary conditions: grid and refinement features are shown for the preflood variant WI_1_ (purple area) and postflood variant WI_2_ (orange area). All other model features are identical: surface model boundaries (blue surfaces) are nodal Waikirikiri River input (WR) and critical‐depth outflow (O), subsurface model boundaries are constant head boundaries in the BPA (green lines, from shallow piezometers), constant head boundaries in the regional aquifer (black lines, from deep piezometers) and no flow boundaries (red rectangles).

Again, two model variants (WI_1_ and WI_2_) were created by utilizing two different DEMs to generate the surface elevation of the model (see Figure 6). The horizontal grid of WI_1_ is comprised of 9798 nodes and 19,195 elements of variable size, edges were as small as a few meters near the river but as long as 40 m away from it (Figure 6). The grid generated from the postflood DEM used for WI_2_ is comprised of 9744 points and 19,285 elements. Furthermore, different to the previous extrapolation of the BPA downstream boundary condition described in Di Ciacca et al. (2024), two new piezometers (D1 and D2, see Figure 4) were installed close to the downstream limit of our study sites and used as a head boundary condition for both model variants in this study.

### Model Calibration and Simulation Scenarios

For the Wairau River model, we utilized two distinct model simulation periods: a preflood data set from August 11, 2020 to October 29, 2020 and a postflood data set from November 10, 2021 to April 10, 2022. Boundary conditions and model forcings were specified at 15‐min time intervals. For the simulations of both the pre‐ and postflood time period, initial conditions for surface water flow were set to 1 × 10^−4^ m water depth, while initial groundwater levels were derived from steady‐state simulations with Wairau River flow of 120 m^3^/s (mean flow during the chosen simulation periods). The other boundary conditions were set up as specified in the chapter above. The conductivity, porosity, van‐Genuchten, friction and coupling length parameters of the WU_1_ model were calibrated manually. For this process, groundwater level measurements from 21 piezometers during the roughly 3‐month preflood data set were utilized. For these piezometers, RSME values of each piezometer, total RSME and graphs depicting measured and simulated head time series were utilized in an attempt to minimize total RSME while avoiding individual outliers and ensuring adequacy of the groundwater level dynamics. Simulations with the postflood input data set were then used to evaluate the model with the calibrated parameter set, using a mixed set of “calibrated on” and independent piezometers not used in the calibration. Manual calibration resulted in parameter values listed in Table 1. The average RSME for the time series of the 21 groundwater piezometer was 0.22 m with values for the individual piezometers ranging from 0.07 to 0.57 m. The larger errors occurred mostly at the piezometers close to the southern model boundary and are thus influenced by the boundary conditions, as well as the coarser model grid. As the following analyses focus on the braidplain aquifer and the interaction with the area of regional aquifer directly underlying the river, a higher simulation accuracy there was deemed more important. In the Results and Discussion section, we present the results from the simulations of WU_1_ and WU_2_ with the postflood period (November 10, 2021 to April 10, 2022), although all analyses were done for both pre‐ and postflood simulation periods. River flow time series for the simulations were chosen to include a variety of flows. Specific high (February 14, 2022) and low (February 2, 2022) flow days were selected for the analysis in the results and discussion. A sufficient spin‐up time beforehand was excluded to ensure no influence of starting conditions. Furthermore, low flow days were chosen at the tail end of river flow recessions to ensure enough time for the slower groundwater systems to equilibrate.

Note that the presented results mostly focus on relative changes, especially in regards to recharge fluxes. The highly detailed surface representation and full coupling of surface and subsurface flow ensure that the model variants can adequately simulate exchange fluxes on small scales, which are the main focus of this study. In contrast, the models presented here are necessarily simplified in their parameterization of subsurface heterogeneity and description of lateral boundary fluxes due to a lack of data and prohibitive run times. Since these model assumptions always apply to both paired different model variants, their effects on the relative changes presented and discussed below are considered negligible. A comprehensive uncertainty analysis of these effects is beyond the scope of this study and computationally hardly feasible.

For the Waikirikiri River, simulations were conducted for the period between June 2, 2020 and January 31, 2021 (preflood) and the period between October 1, 2021 and October 1, 2022 (postflood). The details of the model forcings and parameterization can be found in Di Ciacca et al. (2024). The analysis presented in results and discussion is based on the postflood inputs, again with two specific high (July 12, 2022) and low (May 7, 2022) flow days chosen.

## Results and Discussion

In the following section, the model variants of Wairau River (WU_1_ and WU_2_) and Waikirikiri River (WI_1_ and WI_2_) models are presented to answer the following questions: Is river–groundwater interaction in braided rivers changing over time? And if so, can changing recharge rates be attributed to changes in water levels, which in turn are affected by (certain) differences in braided river morphology? Changing morphology will be analyzed through comparison of the two model variants based on the different DEMs, labeled pre‐ and postflood, respectively, due to the high flow events separating them.

### Changes in Braidplain Aquifer Recharge and Regional Aquifer Recharge

#### Braidplain Aquifer Recharge

Due to the typical hydrogeological features of braided river–groundwater system explained above, river–groundwater interactions are investigated at two different interfaces within the system. First is the exchange between river and braidplain aquifer, calculated for the subdomains defined in Figures 3 and 4, respectively. Table 2 summarizes the water fluxes between river and braidplain aquifers for both model variants of the two test cases for two different flow regimes. Besides recharge, defined as the net sum of the fluxes entering (positive sign) and leaving (negative sign) the subsurface, the exchange flow is further compartmentalized into BPA inflow (from the river) and BPA outflow (to the river). All values presented are mean daily fluxes over the respective low/high river flow days chosen from the simulated time.

**Table 2:** Comparison of Mean Daily Water Fluxes Between Wairau River/Waikirikiri River and Braidplain Aquifers for Pre‐ and Postflood Morphology Variants Each, Simulated During Both a Low Flow as well as a High Flow Day

		Wairau River			Waikirikiri River		
		WU_1_	WU_2_	Change (abs./rel.)	WI_1_	WI_2_	Change (abs./rel.)
Low flow	BPA inflow, m^3^/(s km)	0.58	0.76	+0.18/+31%	0.34	0.27	−0.07/−21%
	BPA outflow, m^3^/(s km)	0.29	0.36	+0.07/+24%	0.03	0.04	+0.01/+33%
	BPA recharge, m^3^/(s km)	0.29	0.40	+0.11/+38%	0.31	0.23	−0.08/−26%
High flow	BPA inflow, m^3^/(s km)	1.32	1.37	+0.05/+4%	0.39	0.32	−0.07/−18%
	BPA outflow, m^3^/(s km)	0.72	0.70	−0.02/‐3%	0.05	0.07	+0.02/+40%
	BPA recharge, m^3^/(s km)	0.60	0.67	+0.07/+12%	0.34	0.25	−0.09/−26%

Note: Braidplain aquifer recharge is divided into inflow from the rivers, outflow to the rivers and total recharge.

For the Wairau River study site, total BPA recharge is increasing in the postflood morphology variant, with larger changes at low flow. This is in spite of the likewise increase in BPA outflow at low flow conditions, which counteracts the overall BPA recharge increase, as it is overshadowed by the stronger increase in BPA inflow. This means that morphology changes in the Wairau River increase river–groundwater interaction in all flow directions while amplifying the existing BPA recharge signal. At high flow, changes in both in‐ and outflow are small, but both contribute to the increase in total BPA recharge.

In the Waikirikiri River, postflood morphology trends are reversed: for both low and high flow, total BPA recharge is decreasing. While the absolute values are small in comparison to the Wairau River, relative changes show significance of BPA recharge decrease due to Waikirikiri Rivers overall lesser flows. In the Waikirikiri River, the decreases in BPA recharge are driven by both inflow decreases and outflow increases for low and high flow, meaning the morphology changes influence river‐groundwater interaction in a unified direction in this case. Furthermore, differences between low and high flow are small, especially compared to the Wairau River. This can likely be attributed again to the overall lesser flows in the river, leading to less dramatic changes in flow regimes.

#### Regional Aquifer Recharge

Simulated regional aquifer recharge (again, for the subdomains) can be compared both between the pre‐ and postflood model variants of both study sites as well as in regards to the changes in BPA recharge shown above. Due to the differences in regional aquifer connection, regional aquifer water levels close to the Wairau River are very similar to the BPA water levels and both systems are horizontally well connected, while the regional aquifer groundwater head in the Waikirikiri River lies several meters below the base of the BPA and recharge happens through vertical infiltration through the BPA base.

In both Wairau River and Waikirikiri River, the direction and magnitude of change of regional aquifer recharge coincide with the BPA recharge changes shown above, as detailed in Table 3. This means that independent of the connection between BPA and regional aquifer, morphological changes in the river channels can be traced to changes in regional aquifer recharge. Furthermore, the different direction of change (in‐/decrease for Wairau River and Waikirikiri River, respectively) between the two study sites shows that such morphology changes can in‐ or decrease regional aquifer recharge. However, it is important to note that the magnitude of these changes is smaller for the regional aquifer recharge than for the BPA. This is especially the case for the Waikirikiri River, where the changes in regional aquifer recharge are four times smaller than for the BPA.

**Table 3:** Daily Mean of Simulated Total Recharge to Regional Aquifer From Wairau River/Waikirikiri River for Pre‐ and Postflood Morphology Variants Each

	Wairau River			Waikirikiri River		
	WU_1_	WU_2_	Change (abs./rel.)	WI_1_	WI_2_	Change (abs./rel.)
Regional aquifer recharge [m^3^/(s km)]	0.44	0.50	+0.06/+14%	0.33	0.31	−0.02/−6%

#### Summary of Recharge Changes

The effects of the change in river morphology on BPA and regional aquifer recharge are as follows:

- different morphology can increase (Wairau River) or decrease (Waikirikiri River) BPA recharge,
- directions of recharge change (in‐/decrease) to the BPA is matched by the same direction of recharge change to the regional aquifer,
- the magnitude of changes in regional aquifer recharge is smaller than BPA recharge changes.

### Changes in Surface and Subsurface Water Levels

As shown above, changes in braided river morphology after a flood event can both in‐ or decrease simulated regional aquifer recharge under otherwise constant conditions. As recharge is heavily influenced by the interplay between surface and subsurface water levels, this raises the question if those are similarly influenced by the changes in morphology.

#### Groundwater Levels

**Figure 7:**
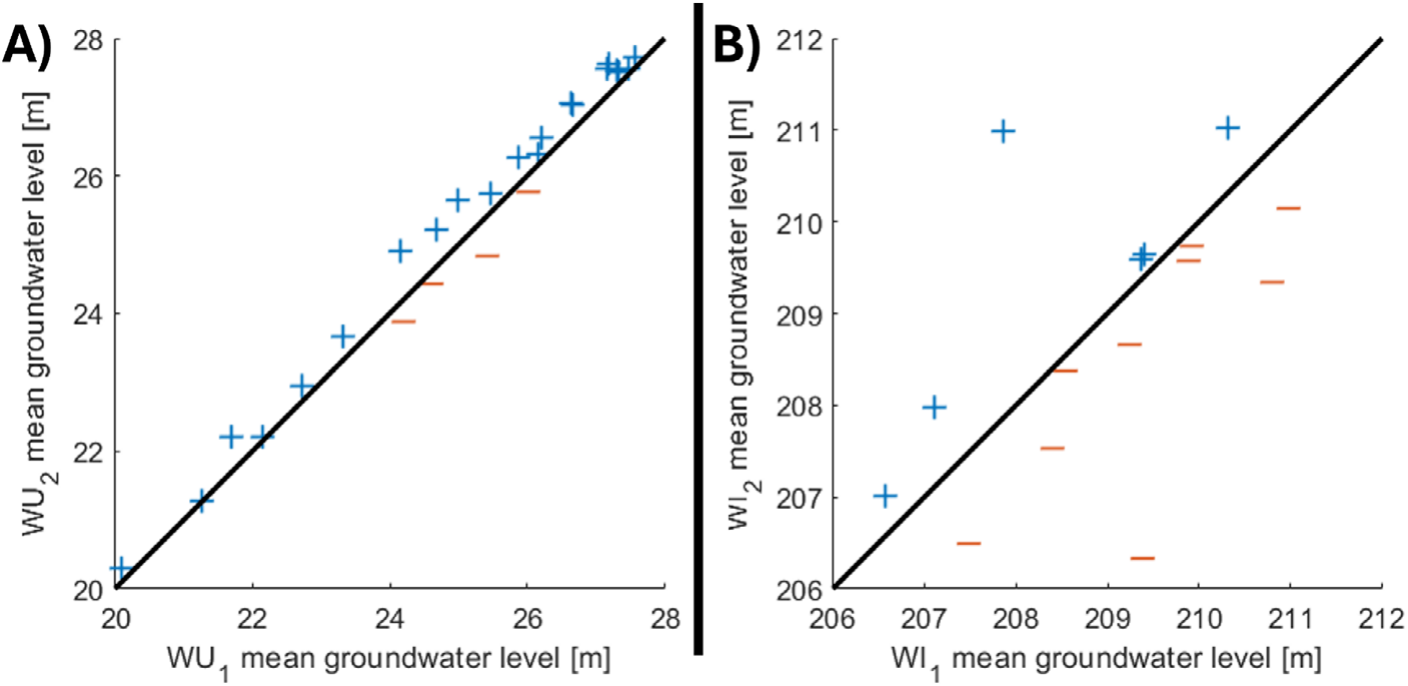
Influence of morphological changes in Wairau River (A) and Waikirikiri River (B) on groundwater levels: simulated mean values of preflood (WU_1_/WI_1_) versus postflood (WU_2_/WI_2_) model variants. “+” indicates rise in groundwater level postflood, while “−” indicates postflood decline.

Due to the differentiation between braidplain aquifer and regional aquifer, regional aquifer recharge is mainly influenced by BPA groundwater levels, and not directly by river water levels (which do influence BPA groundwater levels in turn), as was also observed in Di Ciacca et al. (2024). Figure 7 compares time‐averaged simulated groundwater levels at the piezometer locations (shown in Figures 3 and 4) for both Wairau River and Waikirikiri River for the two model variants.

In the Wairau River, a majority of groundwater levels show an increase in the postflood morphology model variant. The location of most of these measurement points inside the braidplain aquifer explains the increase in regional aquifer recharge that was detailed above: higher BPA groundwater levels lead to an increased gradient, which increases groundwater flow with all other factors, like hydrogeology, being constant.

Similarly, we see a decline of most groundwater levels in the Waikirikiri River in the postflood model variant. Appropriately, regional aquifer recharge decreased in the postflood morphology variant of the Waikirikiri River model, as shown above.

Contrary, the gradient governing BPA recharge is formed by subtracting BPA groundwater levels from river water levels. Thus, in isolation, the direction of BPA groundwater level change described above (increase for Wairau River, decrease for Waikirikiri River) would lead to inverse BPA recharge changes (decrease in Wairau River, increase in Waikirikiri River). Instead, Table 2 above showed that BPA recharge changes agree in direction with the BPA groundwater level changes. As the gradients in question are defined between BPA groundwater levels and river water levels, one can assume that the latter had to change between the morphology variants as well to explain this, as both variables are highly dependent on each other.

#### River Water Levels

**Figure 8:**
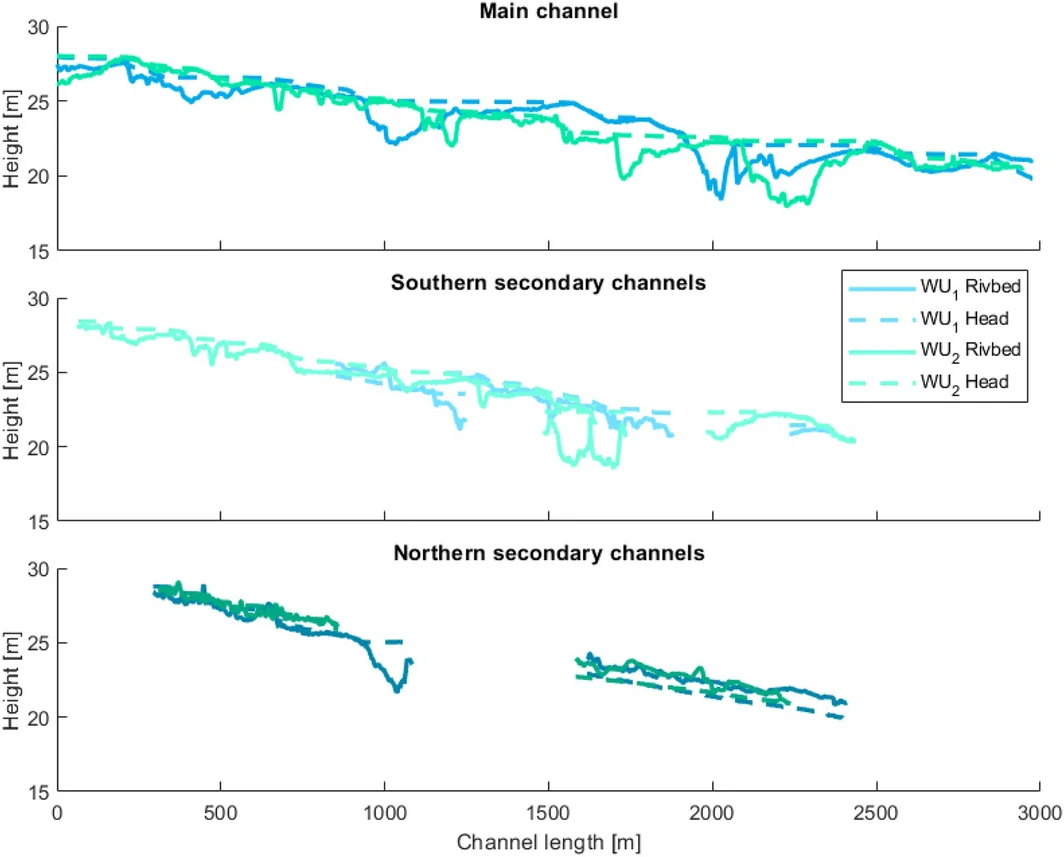
River Head analysis along main and secondary channels for both Wairau River morphology variants, preflood (WU_1_, blue) and postflood (WU_2_, green), for a low flow day. Pictured are channel riverbeds (solid lines) and channel river heads (dashed lines). The locations of the channels can be found, similarly coded, in Figure 3.

Figure 8 shows horizontal profiles of riverbeds and accompanying mean river heads for the two morphology variants of the Wairau River model at a low flow day. Depicted are main channels of the Wairau River, as well as secondary channels in the northern and southern part of the braidplain for both morphology variants. For both variants, riverbed incision in the northern secondary channels is very similar, and the channels are dry during low flow. In the main channel, river heads are slightly higher in the preflood variant in about the first half of the river, while heads are higher in the postflood variant in the downstream part of the river. Riverbed incision is also lower preflood in the upstream half of the river, and overall lower postflood in the downstream section of the river. The southern secondary channel is short and mostly dry in the preflood variant, while it is much longer and generally water‐bearing, even during low flow, in the postflood variant.

While these findings in Figure 8 do not seem to paint a readily conclusive picture regarding river water levels in the Wairau River, assumed to be higher in the postflood variant due to increase of BPA recharge in spite of increased BPA groundwater levels, the location of changes in the braidplain of the Wairau River might further influence these phenomena: the preflood variant exhibits higher river water levels in the channels transitioning from southern bank to northern bank, and along the northern bank, in the first half of the river. In contrast, the postflood variant shows higher river water levels in the later river areas along the southern braidplain bank, and a water‐bearing, long secondary channel along the southern bank. This could be important for river–braidplain aquifer exchange due to the gradient of regional groundwater flow: in the study area, groundwater is mainly flowing along the river, exhibiting higher heads in the west and lower heads in the east, but a second, smaller gradient from north to south exists, with mean groundwater heads declining by about 1.5 m from the northern to southern study edge. This means that gradients between river and BPA groundwater are potentially higher along the southern bank of the braidplain, and thus river channels along this bank are more important for potential recharge to the BPA.

**Figure 9:**
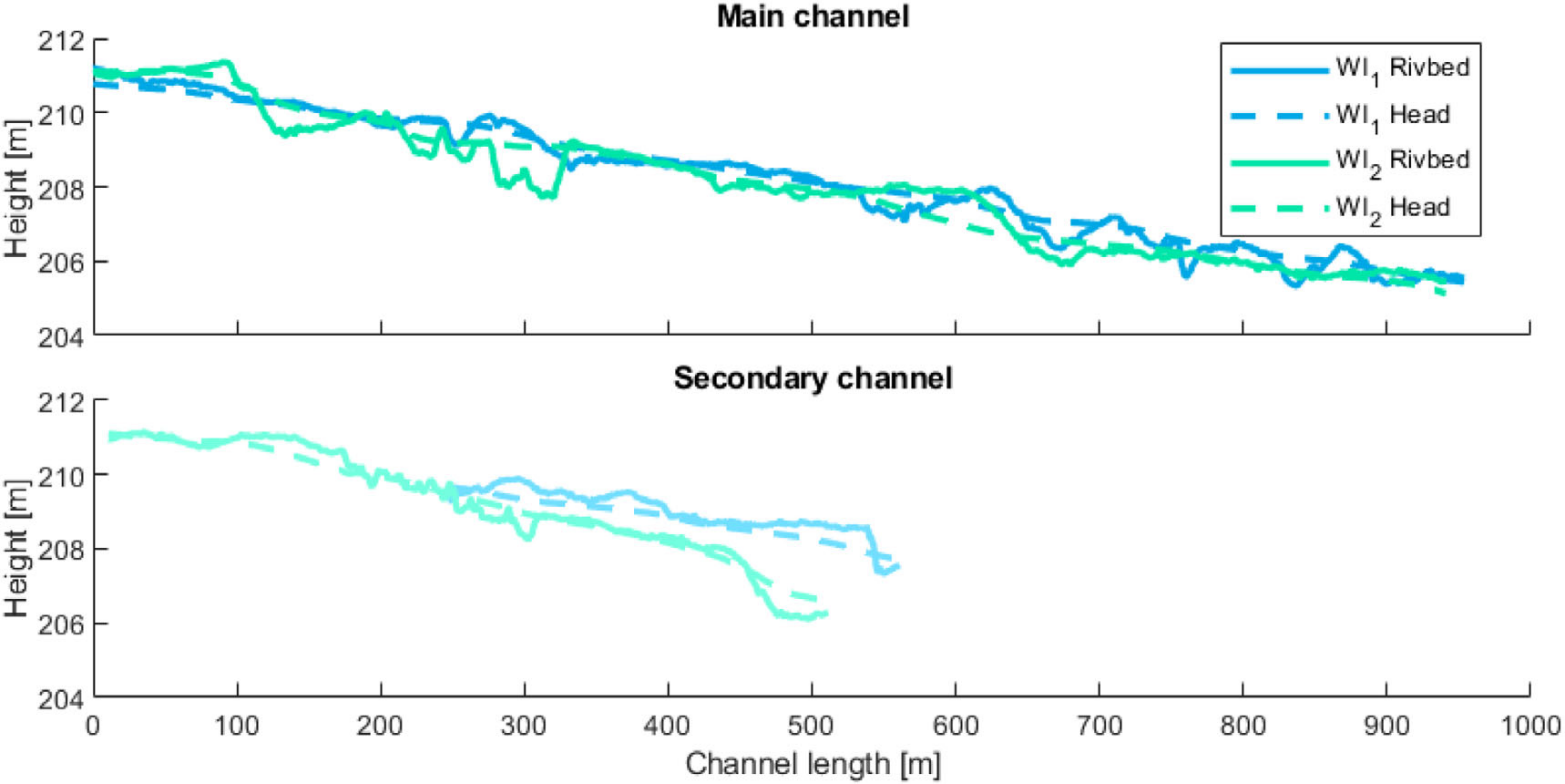
River Head analysis along main and secondary channels for both Waikirikiri River morphology variants, preflood (WI_1_, blue) and postflood (WI_2_, green), for a low flow day. Pictured are channel riverbeds (solid lines) and channel river heads (dashed lines). The locations of the channels can be found, similarly coded, in Figure 4.

Figure 9 depicts riverbeds and mean low flow river heads for the Waikirikiri River model variants in a similar fashion. Due to less braiding in the study area, only a single secondary channel is differentiated from the main channel for each morphology variant. For both variants, the secondary channels are dry during low flow, thus not contributing to exchange with the braidplain aquifer. In the main channel, the postflood variant has several areas of deeper incision, thus allowing a better connection between river and groundwater. Contrary, the river heads in the main channel of postflood morphology variant are significantly lower than for the preflood morphology. This stark decrease in river heads is likely the driver of the reduced BPA recharge of the postflood morphology variant, with less significant decreases in postflood BPA groundwater heads despite their relation.

#### Summary of Changes in Water Levels

Different river morphologies affect water levels in river and groundwater:

- Increase (Wairau River) or decrease (Waikirikiri River) of river water levels leads to in−/decrease in BPA recharge, as shown above.
- This further results in in‐/decreasing (Wairau River/Waikirikiri River) BPA groundwater levels, which in turn affect the changes in regional recharge described above.
- River–groundwater exchange is further changed by providing new locations of high gradient (Wairau River) or deeply incised connections (Waikirikiri River).

### Differences in Braidplain Morphology

While changes in river and groundwater levels agree with the in‐/decrease in regional and braidplain aquifer recharge between pre‐ and postflood model variants in the Wairau River and Waikirikiri River, the question remains whether specific morphology changes can be identified that might explain these effects. In addition, we discuss how these findings can be generalized and transferred to other braided river systems without this extensive analysis conducted here.

#### Local, Small‐Scale River–Groundwater Exchange Areas

**Figure 10:**
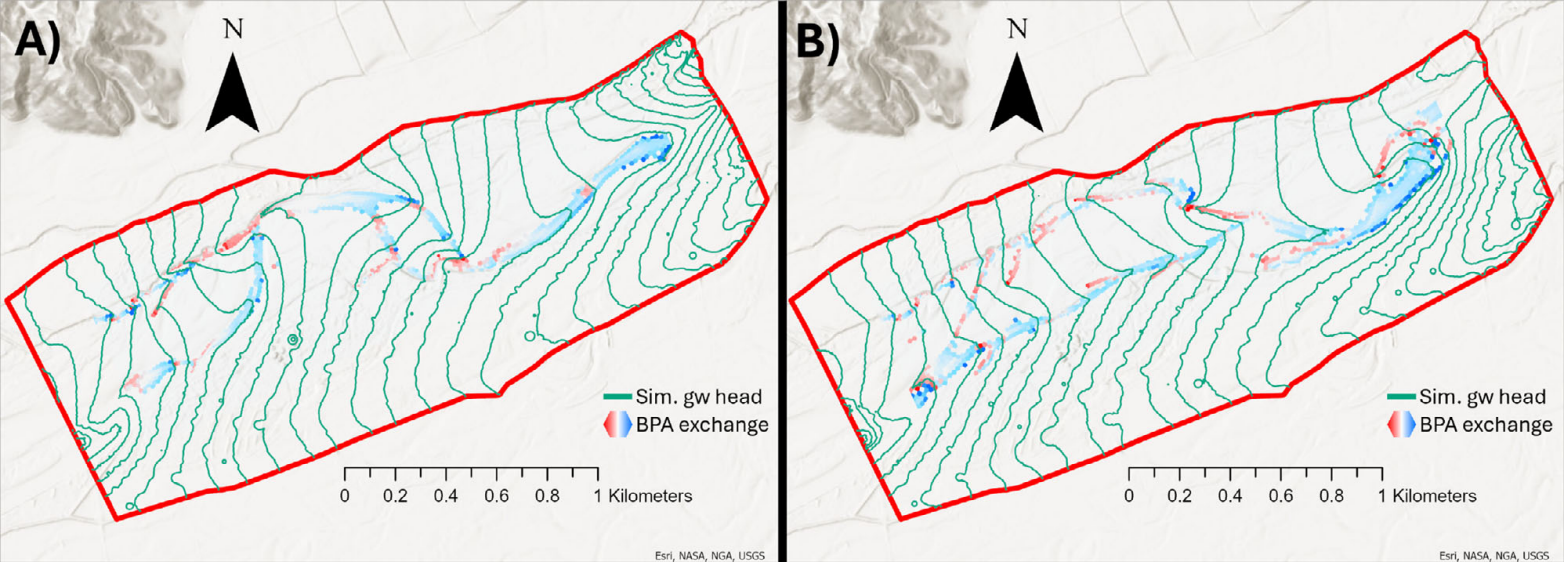
BPA inflow (blue) and outflow (red), as well as simulated groundwater head contours for low flow for preflood WU_1_ (A) and postflood WU_2_ (B) morphology variants for the Wairau River.

A variable indicator of changing river–groundwater exchange between different morphologies could be the strength, location and extent of local, small‐scale river–groundwater exchange areas. Figure 10 shows river–groundwater exchange on a model cell level for the subdomains of the Wairau River pre‐ and postflood morphology variants. While many areas show similar patterns between the two variants, two specific zones are of higher interest, exhibiting clear differences. First, the southern secondary river channel only exists in the postflood variant, and second, the rivers' interaction with the southern braidplain bank in the eastern, downstream part of the model.

The southern secondary channel in the postflood variant was already identified as important in the analysis of river water levels above. Figure 10 shows continuous inflow from the river into the braidplain along this channel, and the local downstream curvature of the groundwater head contours confirms the channel's importance toward recharge. As detailed above, the underlying secondary regional groundwater level gradient from north to south, as seen in the slight south‐eastern tilt of the contours, verifies that the channel's location in the southern part of the braidplain is especially important to its recharge ability.

A second area of clear distinction between pre‐ and postflood morphology variants is the south‐eastern, downstream part of the river. While the postflood model variant shows a few cells of increased outflow from the groundwater to the river in the northern part of this area, this is outweighed by a big inflow band where the river meets the southern bank of the braidplain. While in the preflood model, the river finishes its route from northern bank to southern bank earlier in the study area and flows parallel to the southern bank in the final model part, the postflood variant meets the south‐eastern bank at an angle, being redirected instead of flowing parallel. This seems to enhance BPA recharge, especially due to the lower groundwater heads in this area due to the regional groundwater gradients of west–east and north–south.

In conclusion, the postflood model variant exhibits more exchange‐facilitating local areas (secondary streams, angled bank approaches) in the southern part of the model, congruent to the regional groundwater gradient, thus reinforcing their influence on BPA recharge increase.

**Figure 11:**
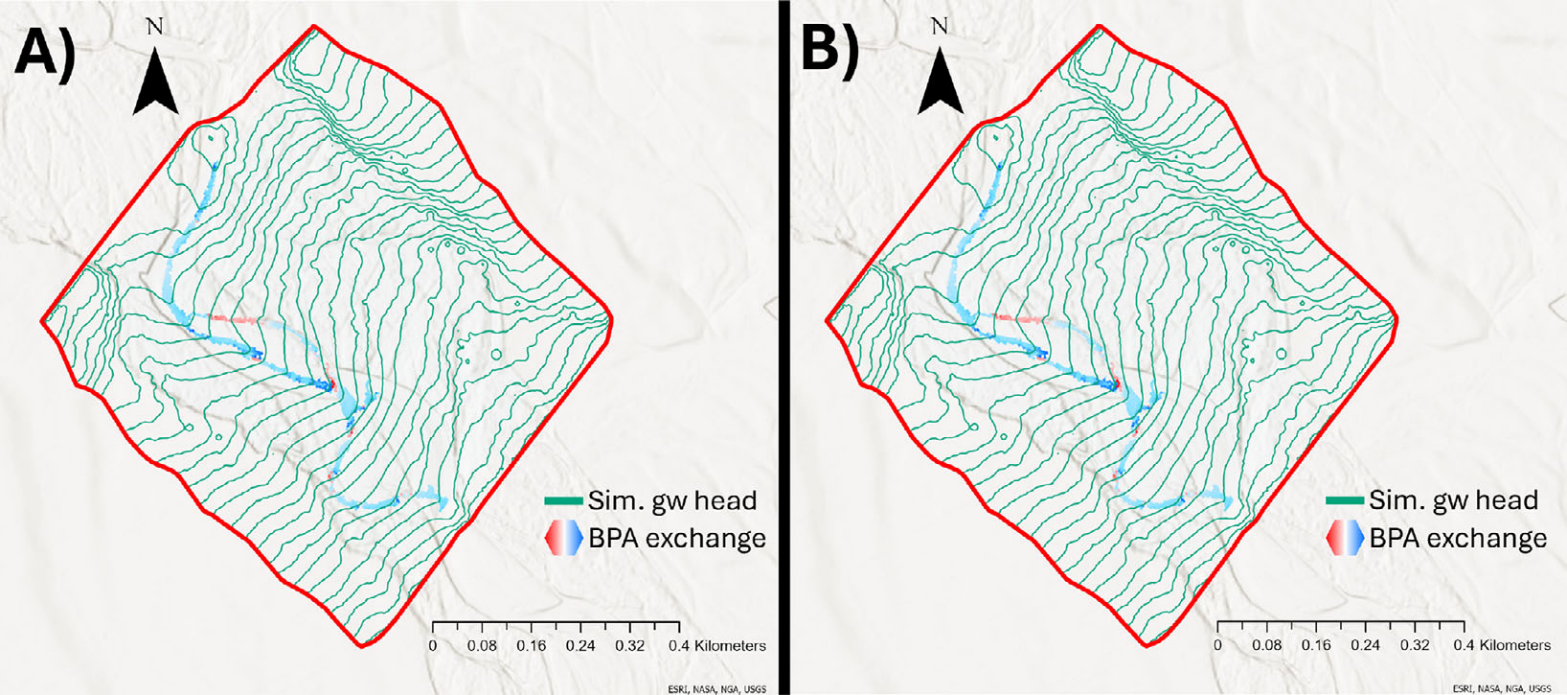
BPA inflow (blue) and outflow (red), as well as simulated groundwater head contours for low flow for preflood WI_1_ (A) and postflood WI_2_ (B) morphology variants for the Waikirikiri River.

In the Waikirikiri River, analysis of local river‐groundwater exchange areas also corroborates the findings from before. The preflood morphology variant, which exhibits higher BPA recharge, shows continuous inflow from river to groundwater along the main river channel, with strong areas in the middle part of the study area (Figure 11). While the postflood model variant also shows almost continuous inflow along its main river channel, it does exhibit a small but significant outflow area after the middle portion of the main channel. Furthermore, the diversion of the main channel away from the western bank in the first part of the study area seemed to open up this secondary channel as an area of outflow from groundwater to river while simultaneously losing the strong inflow area in the middle part. Thus, the diversion of the main channel away from the western and south‐western model banks could be a morphological factor in BPA recharge decrease in the Waikirikiri River.

#### Wetted Surface Areas

Besides gradients, another potentially important factor in river–groundwater exchange in‐/decrease is the wetted area of the river, restricting the surface available for possible exchange. Table 4 summarizes mean daily wetted areas of the subdomains for four different flow conditions, two low and two high flow, for pre‐ and postflood morphology variants of both study areas. In both the Wairau River and Waikirikiri River, the in‐/decrease of wetted river area agrees with the in‐/decrease of BPA recharge, meaning higher postflood wetted areas in the Wairau River, where BPA exchange was also higher, and reductions of both wetted areas and BPA recharge in the postflood variant of the Waikirikiri River model. For high flow conditions, the same holds true for the Waikirikiri River, while for the Wairau River, wetted areas do not change significantly in high flow conditions. This might indicate that the flood event dividing the two morphology variants did not strongly alter the higher‐elevation portions of the braidplain activated during high flows.

**Table 4:** Changes in Mean Daily Wetted Area for Four Flow Conditions Between Pre‐ and Postflood Morphology Models for Both Wairau River and Waikirikiri River

	Wairau River			Waikirikiri River		
Flow Conditions and Wairau River/Waikirikiri River Flow [m^3^/s]	WU_1_ [m^2^]	WU_2_ [m^2^]	Change [%]	WI_1_ [m^2^]	WI_2_ [m^2^]	Change [%]
Low: 11/0.43	109,511	156,287	+42.7	14,742	12,023	−18.4
Low: 64/0.47	212,460	244,623	+15.1	12,595	12,214	−3.0
High: 308/10.19	318,138	317,213	−0.3	18,677	15,005	−19.7
High: 635/30.67	350,311	343,500	−2.0	36,264	26,879	−25.9

#### Braidplain Aquifer Elevation and Volume

**Figure 12:**
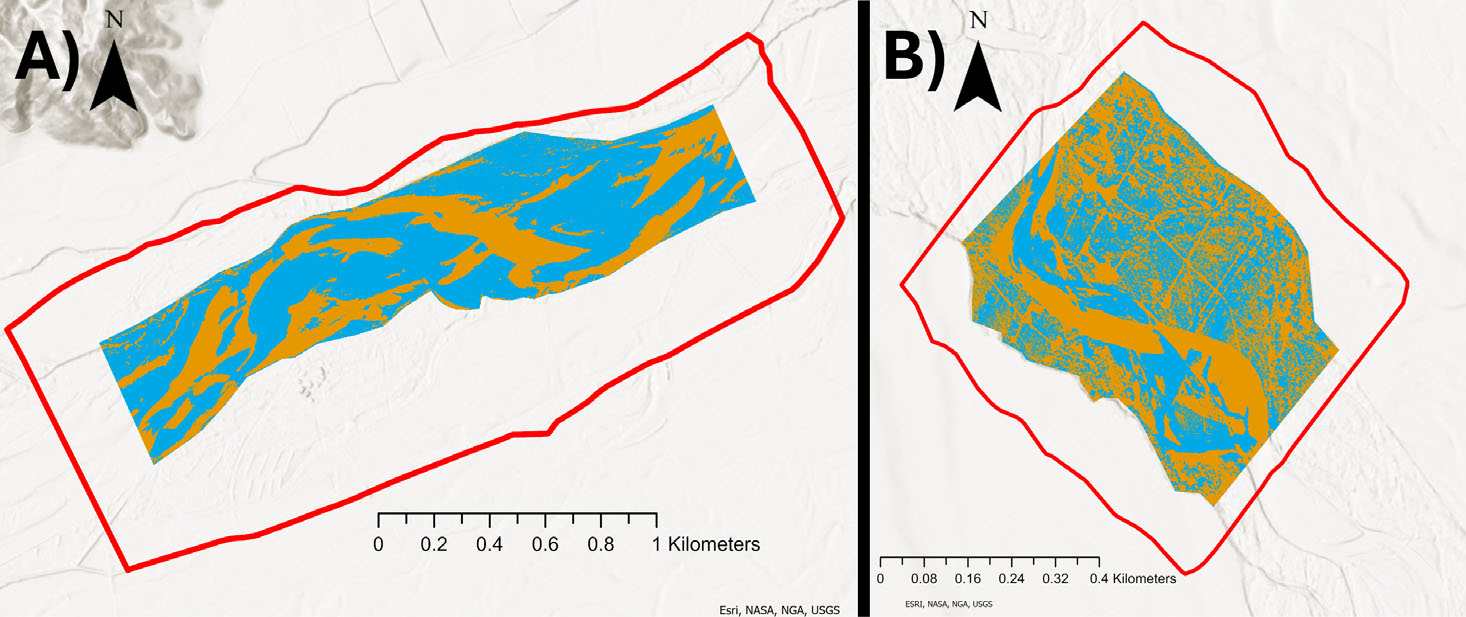
Areas of braidplain volume change between the pre‐ and postflood morphologies, where blue is an increase for the postflood variant, and orange is a decrease, for Wairau River (A) and Waikirikiri River (B). For the Wairau River, the braidplain aquifer shows an increase of about +83,500 m^3^ (volume)/+0.08 m (mean elevation) for the postflood variant, while for the Waikirikiri River a decrease of about −43,300 m^3^ (volume)/−0.15 m (mean elevation) for the postflood variant is calculated.

Besides the different hydrological influences of morphological changes due to major flood events discussed above, reworking of the braidplain also alters elevation and volume of the braidplain aquifer, thus potentially changing the hydrogeological situation. Figure 12 shows areas of braidplain volume and elevation change of the subdomains for both Wairau River and Waikirikiri River calculated from the pre‐ and postflood DEMs. While small, changes in mean braidplain aquifer elevation for both study areas agree with the in‐/decrease in BPA and regional aquifer recharge: for the Wairau River, braidplain aquifer elevation increased in the postflood variant, while for the Waikirikiri River a decrease in mean BPA elevation is registered. Physically, the mean braidplain elevation can be linked to river water and BPA groundwater levels, thus playing a major morphological role in the changes described above. Similarly, while the distribution of volume in‐/decrease in space is not really informative, the total volume changes in both study sites agree with the hydrogeological changes described above as well. Higher braidplain aquifer volume allows more BPA groundwater storage, which could lead to higher regional aquifer recharge due to a more stable exchange regime. Furthermore, more braidplain aquifer volume probably allows for more possible local river‐groundwater exchange areas, thus facilitating higher BPA recharge as well.

#### Summary of Braidplain Morphology Differences

Analysis of the morphological changes of the braidplain support the findings on water levels and exchange by:

- further clarifying the importance of changes in local, small‐scale exchange areas like angled banks or secondary channels,
- illustrating that in‐/decrease of wetted area under low flow coincide with the directions of change in recharge in both study sites,
- and showing that BPA groundwater storage, important for regional aquifer recharge, is changing with the volume of braidplain material sediments deposited or eroded.

### Overall Summary

We presented changes of hydrogeological, hydrological, and morphological factors derived from modeling changed surface morphology from a singular flood event in two study sites under otherwise constant conditions regarding hydro(geo)logical parameters and boundary conditions. Under the lens of river–groundwater interaction, the direction of recharge change was different for the two study sites, with an increase in both BPA and regional aquifer recharge in the postflood variant for the Wairau River, and a decrease in both recharge types in the postflood Waikirikiri River.

The similarities in the postflood changes for both study sites can be summarized as follows:

- The two case studies show changes in braidplain aquifer elevation and volume: an increase in the Wairau River, a decrease in the Waikirikiri River.
- These changes directly relate to changes in wetted area, river and groundwater levels, which are consistent for both study sites: increasing for Wairau River, decreasing for Waikirikiri River.
- Changing river levels directly influence the gradients to BPA levels, leading to matching in‐/decrease in Wairau River/Waikirikiri River BPA recharge rates.
- BPA recharge is further affected by accompanying changes in local inflow zones inside the braidplain and along the banks.
- In‐/decrease in BPA groundwater levels result in similar but smaller in‐/decrease in regional aquifer recharge for Wairau River/Waikirikiri River, respectively.

The correlation between the changes to braidplain elevation and aquifer volume, river water levels, BPA groundwater levels and recharge fluxes (to the BPA and to the regional aquifer) is well explained physically. Higher topographic elevation of the braidplain usually leads to higher river levels, which in turn result in higher groundwater levels in the BPA, higher gradients and thus higher fluxes. However, the effect of river level changes on regional recharge magnitude depends, among other factors, on the relative importance of this change compared to the overall gradient. In connected systems, like the Wairau River, river level changes might significantly in‐/decrease the gradient, while in disconnected systems, like the Waikirikiri River, similar river level changes will only slightly change the overall gradient, as shown in the analysis above. A further, directly physically linked factor is wetted areas, which are positively correlated to BPA recharge as shown in the results of this study. This might be further enhanced by the direct morphological changes to the river networks, evident through the change in existing local inflow zones. Furthermore, braidplain aquifer volume changes also showed to be in alignment with the recharge changes, probably by altering the available groundwater storage and possibly playing a role in the formation of local inflow zones as well.

The discussion above focused on the similarities between the two study sites in their agreement regarding the direction of change of braidplain elevation and volume, wetted area, water levels and recharge fluxes. A clear distinguishing factor between the two study sites is this direction of change: for the specific section of the Wairau River, the aftermath of this flood event saw an increase in levels, fluxes and morphological indices, while for the Waikirikiri River site those indices decreased after the flood. While the changes in hydrogeology are, as described above, driven by the morphological changes brought by these flood events, the reasons for the contrasting morphological responses of the two study sites to their respective floods cannot be clearly identified. Out of scope of this study, antecedent conditions in hydrology and geology, the magnitude of the flood and further features all influence the direction of change brought by the flood. In a flood, some river sections might show aggradation while others are degraded/eroded as part of the natural sediment flow across river plains.

The relationship between the morphological/hydro(geo)logical features with and corresponding changes in recharge have been found in both study sites, despite their different settings with respect to their connection (Wairau River) or disconnection (Waikirikiri River) between BPA and the regional aquifer. This demonstrates that the state of connection is not changing the overall dependencies of recharge on factors such as water levels, wetted area, etc. described above, while clearly influencing the magnitude of changes, as discussed above. These physical dependencies allow some generalization of our findings from the two case studies.

Regular surveys of high‐resolution riverbed elevations allow a simple analysis of BPA volume changes, river channel locations and riverbed levels, which all correlate with recharge changes. These surveys can also be used to derive river water levels and wetted areas with hydraulic modeling tools. The timing of new surveys could be determined by monitoring changes of river channel locations from (publicly available) aerial photographs or by field visits. This information can be complimented by an analysis of river and groundwater level observation time series before and after flood events. In particular, changes in mean BPA water levels would be a strong indication for changes of regional groundwater exchange. An in‐/decrease would indicate a corresponding change in regional aquifer recharge, although a quantitative assessment of the change would not be so easy. However, these general findings and interdependencies are useful for evaluating management options in rivers with competing uses (e.g., recharge source, gravel extraction, flood mitigation). The existence of these simpler indicators could further be used for preliminary quantification of recharge changes with less complex models (see Di Ciacca et al. (2024) for such a study on the Waikirikiri River).

Sai Louie et al. (2025) quantified inter‐annual variations in groundwater fluxes within the braidplain aquifer of the Waikirikiri River using active‐distributed temperature sensing collected from two fiber optic cables. Our findings are in agreement with their general conclusion that flood events, which are reshaping river morphology, can considerably change river–groundwater exchanges in braided river systems. However, Sai Louie et al. (2025) investigated different scales to our study. Furthermore, our study focuses on isolating the effect of river topography on river–groundwater exchanges by comparing model simulations with different surface grids but the same boundary conditions, while Sai Louie et al. (2025) observed the changes caused by concurrent changes in river topography and flow conditions. A formal comparison of the results from active‐distributed temperature sensing and the HGS model outputs could be considered in future work, but would represent a considerable effort in order to make the observations and model outputs comparable.

Nonetheless, the study showed that indicators of in‐/decrease of BPA and regional aquifer recharge can be detected with relatively simple measures, based on elevation surveys and water level time series, without the need for extensive modeling. Therefore, while quantification of groundwater recharge through braided river systems is highly complex and not feasible in most management scenarios, estimation of the existence of flood‐induced changes of recharge due to morphological factors is possible with lesser expenditure. Despite that, thorough quantification necessitates complex models. Unfortunately, applying these complex models at the catchment/regional scale is not currently feasible due to computational and data requirements. The representation of such complexity in simpler models that can be applied at the catchment/regional scale for water management studies still requires further improvements, notably on the relationship between river and BPA levels.

## Conclusions

In this study we demonstrated that flood‐induced morphology changes in braided river systems influence groundwater recharge, both between the river and the braidplain aquifer as well as between the braidplain and the regional aquifer system. We also illustrated that the exchange in the “river–BPA–regional aquifer” system is, while highly connected and interdependent, two‐tiered and needs to be implemented and analyzed separately. As expected, the changes in mean recharge rates correlate with changes in river water and groundwater levels, wetted surface area and existence and location of local inflow zones, but also other features such as braidplain aquifer volume and river channel location within the braidplain. Some of these features can be monitored or estimated without the need for extensive modeling, thus allowing the qualitative estimation of morphology‐induced recharge changes. These findings were derived for both connected and disconnected regimes between braidplain and regional aquifer as represented on our two case studies. Despite the progress made, further research is necessary to strengthen the connections between the different hydro(geo)logical and morphological features and the change to recharge. Also, different areas of braided river systems, such as gaining streams, could be analyzed in this regard as well. Upscaling to longer reaches of rivers is another interesting aspect that is still unclear. This could further help identify patterns in the direction of change and their possible causes, as would the analysis of further morphology variants after several flood events to analyze changes in behavior at the selected sections of the two rivers analyzed here. Finally, correlations that might allow (rough) quantification of change of recharge through other, simpler to gain measures are probably hard to estimate, but could be very valuable.

## Authors' Note

The authors do not have any conflicts of interest or financial disclosures to report.

## Data Availability

The data that support the findings of this study are available from the corresponding author upon reasonable request.
